# Nigerian Children with Acquired Heart Disease: The Experience in Lagos

**Published:** 2017-10

**Authors:** Barakat Adeola Animasahun, Akpoembele Deborah Madise-Wobo, Olusola Yejide Kusimo

**Affiliations:** *Department of Paediatrics and Child Health, Lagos State University College of Medicine, Ikeja, Lagos, Nigeria*

**Keywords:** *Cardiovascular diseases*, *Diagnosis*, *Pediatrics*, *Nigeria*

## Abstract

**Background:** Most of the recent reports on acquired heart diseases (AHDs) among Nigerian children are either retrospective or cover a short period of time with fewer subjects. The last report on AHDs among children in Lagos was about a decade ago; it was, however, not specific to children with AHDs but was part of a report on structural heart diseases among children in Lagos. The present study was carried out to document the prevalence and profile of different AHDs in children and to compare the findings with those previously reported.

**Methods:** We conducted a quantitative, nonexperimental, prospective, and cross-sectional review of all consecutive cases of AHDs diagnosed with echocardiography at the Lagos State University Teaching Hospital between January 2007 and June 2016. Comparisons between the normally distributed quantitative data were made with the Student t test, while the χ^2^ test was applied for the categorical data.

**Results:** The subjects with AHDs were 73 males and 52 females, with a male-to-female ratio of 1.4:1. The children were aged 15 days to 14 years, with a mean of 6.61 ± 4.26 years. Rheumatic heart disease was the most common AHD, documented in a quarter of the children, followed by dilated cardiomyopathy and pericardial effusion in 20.8% and 17.3%, respectively. Less common lesions encountered were Kawasaki disease, mitral valve prolapse, hyperdynamic circulation, and supraventricular tachycardia.

**Conclusion:** Rheumatic heart disease was still the most common AHD in the children in the present study. Dilated cardiomyopathy and pericardial effusion are on the increase as has been reported earlier.

## Introduction

Acquired heart diseases (AHDs) are a heterogeneous group of disease entities affecting the heart and great vessels of children. AHDs are responsible for significant morbidity and mortality in children.^1^^, ^^2^ There are various types of AHDs, including rheumatic heart disease (RHD), Kawasaki disease, cardiomyopathies, pericarditis, and cor pulmonale. The prevalence of the AHDs in children varies from region to region. In Nigeria, the rates are between 28.1% and 68 %.^3^^–^^5^

The prevalence of the various types of AHDs differs among geographical regions and even within similar geographic regions. Four to 5 decades ago, RHD was the predominant type of AHDs worldwide but with improvement in living conditions, availability of antibiotics such as penicillins, and access to health facility, there has been a reversal in that trend in developed countries (report of WHO expert consultation, Geneva, October to November 2001). For example, in the United States of America, Kawasaki disease has replaced RHD as the most common AHD.^6^ In developing countries, RHD was also the most common a few decades ago, but the trend seems to be changing as reported by some authors.^3^^, ^^7^ In more recent studies in Nigeria, different authors have reported varied prevalence rates for AHD types.^3^^, ^^7^^, ^^8^ In South Western Nigeria, Okoromah et al.^3^ reported that pericardial effusion was the most predominant type, followed by RHD and myocarditis. Sadoh and his co-authors^9^ also reported myocarditis and cardiomyopathies as the commonest. In contrast, Sani, Ahmed, and Jiya,^10^ in North Western Nigeria, reported RHD as the most common. Similarly, in a recent report by Nkoke et al.,^11^ from Cameroon, RHD was the common AHD. Thus, there are variations in the types of AHDs amongst regions. The question is why there are variations in the types of AHDs even in Nigeria. Given that RHD is associated with poor living conditions, there is a possibility that there are improved living conditions in some regions with a direct effect on the prevalence of RHD. Echocardiography has been readily available and widely used in recent times, making it possible to swiftly diagnose various AHDs; this may have been responsible for the higher prevalence of the other types of AHDs.

Most of the recent reports on AHDs among Nigerian children are either retrospective reviews of records or have study durations of less than 3 years and involve an insufficient number of subjects. The last report among children in Lagos was about a decade ago; nonetheless, it was not specific to children with AHDs but was part of a report on structural heart diseases among children in Lagos. A prospective review of cases with a longer study period may confer a different result. Also, there are no reports on AHDs in Southern Nigeria. The present study was designed to document the spectrum and clinical characteristics of AHDs in children over a period of 9.5 years. The findings were compared with recent reviews to report changes, if there were any. 

## Methods

The present study was prospective, descriptive, quantitative, and cross-sectional and involved consecutive children aged less than 14 years referred for cardiac evaluation in the Department of Pediatrics of the Lagos State University Teaching Hospital from January 2007 to June 2016. The hospital receives referral within the state and from the adjoining south western states. All the subjects had a detailed history taking with physical examination done. Subsequently chest radiograph, electrocardiography, and echocardiography were conducted. Transthoracic echocardiography was performed on all the subjects using a GE Vivid Q echocardiography machine (reference # 14502 WP SN 2084). This machine had the facility for 2D, M-mode, and color-flow Doppler imaging. One pediatric cardiologist performed the echocardiography on all the patients. 

The echocardiographic diagnoses of the various AHDs were based on the standard diagnostic criteria. The diagnosis of RHD was made in accordance with the World Heart Federation’s guidelines for the echocardiographic diagnosis of RHD.^10^ The diagnosis of cardiomyopathies was also made based on the standard definitions.^11^^, ^^12^ Cor pulmonale and pericardial effusion were diagnosed based on the standard guidelines and diagnosis.^13^

All children with echocardiographic diagnoses of AHDs whose parents granted informed consent were included in the study, and all children with congenital heart lesions and children whose parents refused to grant consent were excluded. 

The variable outcomes were comprised of the type of AHDs, prevalence of the subtypes of AHDs, mean age of the children, and clinical presentation of/indication for cardiac evaluation. 

Ethical clearance for this study was obtained from the Research and Ethics Committee of the Lagos State University Teaching Hospital, and informed consent was obtained from the parents or caregivers of the children. Assent was also taken from children aged 7 years or above.

A structured closed-ended questionnaire was used to obtain necessary information from the subjects and their caregivers. The data were analyzed using Epi Info and SPSS, version 20. Test of normality was assessed using the Kolmogorov–Smirnov tool for normality. Mean and standard deviation were used to summarize the details of the data that were normally distributed. Comparisons of the categorical data were made using the χ^2^ test, while the Student t test was utilized to compare the quantitative data.

## Results

A total of 1,767 echocardiographic examinations were performed in the study period. Congenital heart diseases were diagnosed in 1,180 children and AHDs in 125. Table 1 shows the demographic characteristics of the patients. The subjects with AHDs were comprised of 73 males and 52 females, with a male-to-female ratio of 1.4:1. The children were aged between 15 days and 14 years, with a mean of 79.40 ± 51.13 months (6.61 ± 4.26 y) and median age of 7.6 years. The age of the males and females (mean ± SD) was 80.56 ± 49.22 months (6.71 ± 4.10 y) and 77.78 ± 54.16 months (6.48 ± 4.51 y). There was no significant difference in the age between the 2 genders (p value = 0.775). The modal age subgroup was composed of children aged between 5 and 10 years. 

The most common reason for cardiac evaluation was a presumptive diagnosis of an acyanotic congenital heart disease from the subjects’ history and physical examination; it was documented in over a quarter (26.4%) of the patients. This was followed by congestive cardiac failure and features of RHD/acute rheumatic fever. The other indications are depicted in Table 2. Some of the patients had more than 1 indication for cardiac evaluation. RHD was the most common type of AHDs in that it was documented in a quarter of the patients. This was closely followed by dilated cardiomyopathy (DCM) and then pericardial effusion (Table 3).

**Table 1:** Demographic characteristics of the patients*

Variables	
Gender	
Male	73 (58.4)
Female	52 (41.6)
Age (y)	6.61±4.26
Age groups	
≤ 1 y	23 (18.4)
> 1 y to ≤ 5 y	24 (19.2)
> 5 y to ≤ 10 y	45 (36.0)
> 10 y	27 (21.6)
Missing	6 (4.8)
Median	7.6

* Data are presented as mean±SD or n (%).

**Table 2:** Indications for cardiac evaluation of the subjects*, **

ACHD	33 (26.4)
CCF	20 (16.7)
ARF/RHD	18 (11.7)
Adenoidal hypertrophy	18 (11.7)
Severe LRTI	5 (4.2)
Murmur	6 (3.3)
Chest pain	4 (3.3)
Breathlessness	5 (2.5)
Cyanosis	2 (1.7)
SLE	4 (3.3)
Cancers	3 (2.5)
Cleft lip/Palate	3 (2.5)
Others	18 (13.3)

ACDH, Acyanotic congenital heart disease; CCF, Congestive cardiac failure; ARF, Acute rheumatic fever; RHD, Rheumatic heart disease; LRTI, Lower respiratory tract infection; SLE, Systemic lupus erythematosus; Others, Failure to thrive, hypertension, tachycardia, Down syndrome, retroviral disease, pericardial effusion, and post-cardiac surgery complications

* Data are presented as n (%)

**Most patients had more than 1 reason for cardiac evaluation.

RHD was the most common AHD type; it was documented in a quarter of the children with AHDs. There were 20 males and 12 females, with a male-to-female ratio of 1.7:1. The children were aged between 4 and 13 years, and only 2 children were less than 5 years of age. Most of the patients (50%) were between 5 and 10 years of age at diagnosis, with a mean of 9.41 ± 3.01 years. Most of the patients (63.6%) presented with clinical features in keeping with congestive cardiac failure and RHD. 

Cardiomyopathy was diagnosed in 36 patients, 26 (72.2%) of whom had DCM. DCM was the second most common AHD accounting for 20.8% of all the AHDs. The patients with DCM were 12 males and 14 females with an equal male-to-female ratio. The children were aged between 15 days and 13 years, with mean age of 63.89 ± 48.40 months (5.32 ± 4.03 y) and a median age of 6.25 years. Most of the children (53.8%) were less than 5 years of age, and only 3 (11.5%) were over 10 years of age. Congestive cardiac failure was the most common mode of presentation amongst the subjects with DCM. The remaining children presented with upper respiratory tract infection, generalized edema, tachycardia-induced cardiomyopathy, palpitations, advanced retroviral disease, breathlessness, and cardiomyopathy following the use of Adriamycin for nephroblastoma. One patient also presented with burns cardiomyopathy.

There were 5 cases of restrictive cardiomyopathy accounting for 4% of all the AHDs. There were 2 males and 3 females, aged between 7 and 13 years, at a mean age of 10.00 ± 2.94 years and a median age of 11 years. All the children with restrictive cardiomyopathy had endomyocardial fibrosis.

Hypertrophic cardiomyopathy was diagnosed in 5 patients, accounting for 4.0% of the AHDs. There were 2 males and 3 females, with a male-to-female ratio of 1:1.5. The subjects were aged 3 months to 10 years 7 months, at a mean age of 5.05 ± 4.96 years. Two of the subjects were infants: 1 was 3 years old and the remaining 2 were aged at least 10 years. 

**Table 3:** Gender and age distribution of the different types of acquired heart diseases

	n (%)	Gender		Age (y)				
		Male	Female	≤ 5	< 5 to ≥ 10	> 10	Mean±SD	Median
Rheumatic heart disease	32 (25.6)	20	12	2	16	13	9.49±3.01	10.2
Dilated cardiomyopathy	26 (20.8)	1	14	14	9	3	5.32±4.03	6.3
Restrictive cardiomyopathy	5 (4.0)	2	3	0	2	3	10.00±2.94	11.0
Hypertrophic cardiomyopathy	5 (4.0)	2	3	3	0	2	5.04±4.96	5.7
Cor pulmonale	12 (9.6)	6	6	7	5	0	4.27±3.54	2.7
Pulmonary artery hypertension	8 (6.4)	5	3	3	0	5	3.2±4.9	2.6
Pericardial effusion	19 (15.2)	8	11	9	9	1	6.12±4.0	7.3
Mitral valve prolapse	5 (4.0)	4	1	0	2	3	9.65±2.85	8.3
Hyperdynamic circulation	4 (3.2)	3	1	2	1	1	5.88±4.66	7.6
Kawasaki disease	2 (1.6)	1	1	2	0	0	2.10±2.80	3.0
Others^*^	8 (6.4)	5	3	3	3	2	-	-

Isolated pericardial effusion was documented in 19 (17.3%) of the study subjects. There were 8 males and 11 females, with a male-to-female ratio of 1:1.4. The children were 4 to 13 years old, at a mean age of 6.12 ± 4.00 years. The modal sub-age at diagnosis was between 5 and 10 years. Of the 19 patients, 5 had systemic lupus erythematosus, 3 had Down syndrome, 3 had pulmonary tuberculosis, and 2 had non-tuberculosis pneumonia; the remaining 6 patients had a suspicion of an acyanotic congenital heart disease necessitating cardiac evaluation. 

The pulmonary vascular diseases noted in the study subjects were cor pulmonale and pulmonary artery hypertension. Cor pulmonale was diagnosed in 12 (10.9%) patients with AHDs. All the children with cor pulmonale were less than 10 years of age and the majority of them (63.6%) were less than 5 years old, at a mean age of 4.37 ± 3.54 years. Males and females were equally represented. The most common indication for cardiac evaluation in the study subjects was adenoidal hypertrophy, which was observed in a third of the patients. Two patients, aged 7 and 8.5 years, had been referred for routine pre-surgery cardiac evaluation for cleft lip and palate, respectively, and cor pulmonale was noted. The other patients presented with advanced retroviral disease with marked respiratory distress, failure to thrive, and congestive cardiac failure of an unknown etiology. 

There were 8 patients with pulmonary artery hypertension: 5 males and 3 females. Of the 8 patients with pulmonary artery hypertension, 2 were neonates with persistent primary hypertension of the newborn (PPHN), 1 had severe perinatal asphyxia, and 1 presented with cyanosis and respiratory distress. Of the remaining 6 patients, 4 were children with sickle-cell anemia and the other 2 were toddlers with adenoidal hypertrophy. 

Kawasaki disease was diagnosed in 2 toddlers, aged 19 and 23 months, respectively. Both patients presented with the classic features of Kawasaki disease, including high fever, edema, desquamation of the hands and soles of the feet, cervical lymphadenopathy, non-suppurative conjunctivitis, erythematous skin eruptions over the groin, and ulceration of the lips. Echocardiography revealed coronary artery aneurysm in 1 child and a normal study in the other. Both children responded to oral aspirin and parenteral immunoglobulin. 

Hyperdynamic circulation was noted in 4 patients: 3 males and 1 female. Two of those children were toddlers with sickle-cell anemia and severe nutritional rickets, respectively. The other patients were school-age children with sickle-cell anemia and congestive cardiac failure, correspondingly. 

Mitral valve prolapse was diagnosed in 5 children: 4 males and 1 female. The youngest child was a 4-month-old male with congestive cardiac failure; the others were between 7 and 14 years old with chest pains and persistent cough and generalized edema. 

Supraventricular tachycardia was observed in 2 children, aged 3 months and 2 weeks, respectively. They both had a structurally normal heart with congestive cardiac failure. Their symptoms improved with the treatment of the primary cause of the heart failure. 

Left ventricular hypertrophy was noted in 2 children, aged 9 and 12 years. One had severe hypertension, while the other presented with chest pains. Aortic stenosis co-existing with mitral valve prolapse was observed in 2 patients, both of whom were 10 years of age. 

## Discussion

In recent times, there have been conflicting reports on the prevalence of different AHDs in children. The present study sought to document the spectrum and clinical profile of AHDs in children in a tertiary hospital. The different AHDs documented in the present study in order of frequency were RHD, DCM, pericardial effusion, cor pulmonale, pulmonary artery hypertension, mitral valve prolapse, hyperdynamic circulation, supraventricular tachycardia, left ventricular hypertrophy, mitral stenosis, aortic incompetence, Kawasaki disease, and tumors in the heart.

RHD was the most common AHD, followed by DCM and pericarditis. The distribution of AHDs in the present study mirrors reports from previous studies within and outside Nigeria.^8^^, ^^9^^, ^^14^ However, some recent studies in Nigeria have reported that DCM/myocarditis and pericardial effusion are more common than RHD.^3^^, ^^7^ Earlier studies attributed the low prevalence of RHD, by comparison with the other AHDs, to speculations of improved housing, environmental sanitation, and personal hygiene in the study region and their direct effects on acute rheumatic fever. The reason for the high prevalence of RHD amongst the AHDs compared to the earlier reports is not clear. The difference may be a chance occurrence. Nonetheless, the finding of a higher prevalence of RHD in the present study is not unexpected taking into consideration that RHD has the highest prevalence in the sub-Saharan Africa. To the best of our knowledge, no study has documented a decreased frequency of acute rheumatic fever in sub-Saharan Africa. The implication of this is that RHD is still a problem in the sub-region, and measures to prevent it such as improvement in socioeconomic and environmental conditions and effective primary and secondary preventions for acute rheumatic fever should be put in place.

Cardiomyopathies were the second most common AHD, with DCM being the most common amongst them. The finding in this regard is not surprising in view of the fact that in recent times DCM has been reported as a common cause of AHDs in children,^3^^, ^^7^^, ^^8^^, ^^14^^, ^^15^ even higher than RHD in some studies.^3^^, ^^7^ Diseases that were least recorded such as restrictive cardiomyopathy, hypertrophic cardiomyopathy, and Kawasaki disease were also uncommon in the previous reports in African studies.^3^^, ^^7^^-^^9^^, ^^15^

The children in the present study were aged between 15 days and 14 years, and the majority of them were over 5 years of age. This finding is consistent with reports from previous studies.^7^^–^^9^^, ^^16^ As was expected, age at diagnosis was different with the different AHDs. Most of the children with RHD were over 5 years of age and the majority were between 5 and 10 years old. This finding was consistent with reports from previous studies.^7^^–^^9^^, ^^15^ It is has been shown that RHD is more common in children between 5 and 15 years of age. The children in the present study were within the commonly affected age group. The children with DCM were much younger than those with RHD, and the majority of them were younger than 5 years of age. This was not surprising taking into account that DCM is more common in younger children.^7^^, ^^16^^–^^18^ The mean age of the children with restrictive cardiomyopathy was similar to that reported by previous studies in Africa.^8^^, ^^11^ In contrast, age at diagnosis was much higher than that reported by previous studies in developed countries.^19^ The reason for the older age in the present study and the other African studies may be because of the predominance of endomyocardial fibrosis in the African studies compared to idiopathic restrictive cardiomyopathy commonly reported in the western countries. It has been shown that endomyocardial fibrosis occurs in children in older age groups than in children with idiopathic endomyocardial fibrosis.^20^ The majority of the children with pericardial effusion were over 5 years of age in the present study. This finding was also similar to that reported in previous studies.^8^^, ^^9^ In contrast, in a report by Sadoh et al.,^7^ most of the children were under 5 years of age. The reason for the lower age in the earlier result may be because of the difference in the etiology of pericardial effusion between the earlier study and the present study. In the earlier research, there were more patients with Down syndrome than there were in the present study; this may have resulted in an earlier age at presentation and an overall reduction in the ages of the patients with pericardial effusion. The other diseases that occurred in the children less than 5 years of age in our study were Kawasaki disease, cor pulmonale, and pulmonary artery hypertension. This finding chimes in with the results from previous reports.^7^^, ^^8^

The etiologies of the different cases of AHDs were documented in the present study. RHD is known to follow infection with group A beta-hemolytic streptococci. There was no isolation of the implicated organism in the present study. The etiology of DCM is largely unknown, but genetic and viral infections have been reported.^21^ In the present study, the etiology of DCM was heterogeneous, including tachycardia-induced cardiomyopathy, burns cardiomyopathy, suspected viral myocarditis, anomalous left coronary artery from the pulmonary artery (ALCAPA) syndrome, Adriamycin-induced cardiomyopathy, and HIV infection. Some patients presented with unidentifiable causes. There was no case of suspected or proven muscular dystrophies. Genetic and viral studies could not be done to ascertain the etiology of the patients. Burns cardiomyopathy,^22^^, ^^23^ ALCAPA syndrome,^24^ tachycardia-induced cardiomyopathy,^25^ and Adriamycin-induced cardiomyopathy^26^^, ^^27^ have been reported; they, however, constitute uncommon etiologies of DCM. Few reports on those etiologies have been documented. The patients with restrictive cardiomyopathy in the present study all presented with endomyocardial fibrosis, a finding that is in line with reports from other African studies.^7^^, ^^20^ Pericardial effusion is known to be associated with diverse etiologies. The etiology reported in the present study is in keeping with known results, with a slight difference in the prevalence of the different etiologies.^7^^, ^^8^

Pulmonary vascular disorders documented in the present report were cor pulmonale and pulmonary artery hypertension. Cor pulmonale was the most common pulmonary vascular disorder. The majority of the patients with cor pulmonale had adenoidal hypertrophy. Two patients presented with advanced retroviral disease and respiratory distress, and the etiology was unknown in the other patients. According to previous reports, cor pulmonale has been documented in children with obstructive sleep apnea^28^^, ^^29^ and retroviral disease.^30^ Chronic untreated adenoidal hypertrophy is a common cause of obstructive sleep apnea in children.^31^ It was, therefore, not surprising that a third of our patients with cor pulmonale had adenoidal hypertrophy requiring surgical intervention. It is known that advanced retroviral disease may beget lymphocytic interstitial pneumonia,^32^ which through an unknown mechanism may result in cor pulmonale. Although lymphocytic interstitial pneumonia was not confirmed in our patients, the patients all had advanced HIV/AIDS. The other known etiologies of cor pulmonale such as are pulmonary tuberculosis, asthma, and obesity were not documented in any of our patients. Cor pulmonale left untreated may result in significant morbidity and mortality. It is, thus, important to regularly monitor patients with adenoidal hypertrophy and patients with advanced retroviral disease for cardiac complications such as cor pulmonale so that measures may be implemented early to prevent morbidity and mortality.

The etiology of pulmonary artery hypertension in the present study was related to sickle-cell anemia in half of the patients, and the remaining patients had adenoidal hypertrophy and PPHN. This finding is concordant with the known etiologies of pulmonary artery hypertension in children. There were only 2 cases of PPHN in the present study. Both of these patients were neonates: 1 had severe perinatal asphyxia and the etiology for the second patient was not known. Kawasaki disease was diagnosed in only 2 patients in the current study. The patients were less than 24 months old at diagnosis, and they had the classic presentation of Kawasaki disease. The fact that there were only 2 cases with Kawasaki disease in the present study confirms the postulations that this disease is rare in the sub-region. Another possibility for the low prevalence in the sub-region may be because a high index of suspicion is necessary to make a diagnosis of cases, especially in atypical presentations. Among the previous documentations on AHDs in children in Africa, only a few reports have documented Kawasaki disease.^8^^, ^^9^

The strengths of the present study include its prospective design, reduction in inter-rater bias (bearing in mind that a single cardiologist performed all the echocardiographic examinations), relatively long study period, and relatively large sample size compared to previous studies. Nevertheless, first and foremost among the limitations is that ours is a hospital-based study; thus, only children with severe illnesses presented for evaluation. Some patients may have been referred specifically for cardiac evaluation given that a cardiologist is present in the institution. All those reasons may have resulted in the modification of the disease spectrum seen, which may not be a true reflection of the actual disease burden in the community.

That the present investigation is a single-center study can be considered a limitation. Moreover, it is a study conducted in a tertiary center and hence some patients who could not get to the tertiary center may have been missed. Further, this is a hospital-based study; a community-based study might have been more representative.

## Conclusion

The present study documented the prevalence of various AHDs in a tertiary hospital over a 9.5-year period. Contrary to a recent report, our results demonstrated that RHD was still the most common AHD in the children. The incidence of DCM and pericardial effusion is on the rise as has been reported earlier. A high index of suspicion is required in the diagnosis of other AHDs so as to present data that are more representative. Measures such as improvement in housing and living standards as well as prevention of acute rheumatic fever are still necessary to lessen the burden of RHD.

## Notes:

***This paper should be cited as: ***Animasahun BA, Madise-Wobo AD, Kusimo OY. Nigerian Children with Acquired Heart Disease: The Experience in Lagos. J Teh Univ Heart Ctr 2017;12(4):160-166.

## References

[1] Sliwa K, Mocumbi AO (2010). Forgotten cardiovascular diseases in Africa. Clin Res Cardiol.

[2] Mocumbi AO, Ferreira MB (2010). Neglected cardiovascular diseases in Africa: challenges and opportunities. J Am Coll Cardiol.

[3] Okoromah CA, Ekure EN, Ojo OO, Animasahun BA, Bastos MI (2008). Structural heart disease in children in Lagos: profile, problems and prospects. Niger Postgrad Med J.

[4] Antia AU, Effiong CE, Dawodu AH (1972). The pattern of acquired heart disease in Nigerian children. Afr J Med Sci.

[5] Jaiyesimi F (1982). Acquired heart disease in Nigerian children: an illustration of the influence of socio-economic factors on disease pattern. J Trop Pediatr.

[6] Rowley AH, Shulman ST (1998). Kawasaki syndrome. Clin Microbiol Rev.

[7] Wilson SE, Chinyere UC, Queennette D (2014). Childhood acquired heart disease in Nigeria: an echocardiographic study from three centres. Afr Health Sci.

[8] Sani UM, Ahmed H, Jiya NM (2015). Pattern of acquired heart diseases among children seen in Sokoto, North‑Western Nigeria. Niger J Clin Pract.

[9] Nkoke C, Menanga A, Boombhi J, Chelo D, Kingue S (2015). A new look at acquired heart diseases in a contemporary sub-Saharan African pediatric population: the case of Yaoundé, Cameroon. Cardiovasc Diagn Ther.

[10] Reményi B, Wilson N, Steer A, Ferreira B, Kado J, Kumar K, Lawrenson J, Maguire G, Marijon E, Mirabel M, Mocumbi AO, Mota C, Paar J, Saxena A, Scheel J, Stirling J, Viali S, Balekundri VI, Wheaton G, Zühlke L, Carapetis J (2012). World Heart Federation criteria for echocardiographic diagnosis of rheumatic heart disease--an evidence-based guideline. Nat Rev Cardiol.

[11] Sliwa K, Damasceno A, Mayosi BM (2005). Epidemiology and etiology of cardiomyopathy in Africa. Circulation.

[12] Gajarski RJ, Towbin JA (1995). Recent advances in the etiology, diagnosis, and treatment of myocarditis and cardiomyopathies in children. Curr Opin Pediatr.

[13] Budev MM, Arroliga AC, Wiedemann HP, Matthay RA (2003). Cor pulmonale: an overview. Semin Respir Crit Care Med.

[14] Bode-Thomas F, Ige OO, Yilgwan C (2013). Childhood acquired heart diseases in Jos, north central Nigeria. Niger Med J.

[15] Asani MO, Sani MU, Karaye KM, Adeleke SI, Baba U (2005). Structural heart diseases in Nigerian children. Niger J Med.

[16] Gersh BJ, Maron BJ, Bonow RO, Dearani JA, Fifer MA, Link MS, Naidu SS, Nishimura RA, Ommen SR, Rakowski H, Seidman CE, Towbin JA, Udelson JE, Yancy CW, American College of Cardiology Foundation/American Heart Association Task Force on Practice Guidelines, American Association for Thoracic Surgery, American Society of Echocardiography, American Society of Nuclear Cardiology, Heart Failure Society of America, Heart Rhythm Society, Society for Cardiovascular Angiography, Interventions, Society of Thoracic Surgeons (2011). 2011 ACCF/AHA guideline for the diagnosis and treatment of hypertrophic cardiomyopathy: a report of the American College of Cardiology Foundation/American Heart Association Task Force on Practice Guidelines. Circulation.

[17] Nugent AW, Daubeney PE, Chondros P, Carlin JB, Cheung M, Wilkinson LC, Davis AM, Kahler SG, Chow CW, Wilkinson JL, Weintraub RG, National Australian Childhood Cardiomyopathy Study (2003). The epidemiology of childhood cardiomyopathy in Australia. N Engl J Med.

[18] Antia AU, Cockshott WP, Thorpe GJ (1969). Idiopathic cardiomegaly in Nigerian children. Br Heart J.

[19] Russo LM, Webber SA (2005). Idiopathic restrictive cardiomyopathy in children. Heart.

[20] Sliwa K, Damasceno A, Mayosi BM (2005). Epidemiology and etiology of cardiomyopathy in Africa. Circulation.

[21] Dhandapany PS, Razzaque MA, Muthusami U, Kunnoth S, Edwards JJ, Mulero-Navarro S, Riess I, Pardo S, Sheng J, Rani DS, Rani B, Govindaraj P, Flex E, Yokota T, Furutani M, Nishizawa T, Nakanishi T, Robbins J, Limongelli G, Hajjar RJ, Lebeche D, Bahl A, Khullar M, Rathinavel A, Sadler KC, Tartaglia M, Matsuoka R, Thangaraj K, Gelb BD (2014). RAF1 mutations in childhood-onset dilated cardiomyopathy. Nat Genet.

[22] Chen TJ, Shen BH, Yeh FL, Lin JT, Ma H, Huang CH, Fang RH (2003). Delayed dilated cardiomyopathy for major burn injuries. Burns.

[23] Mak GZ, Hardy AR, Meyer RA, Kagan RJ (2006). Reversible cardiomyopathy after severe burn injury. J Burn Care Res.

[24] Molaei A, Rastkar Hemmati B, Khosroshahi H, Malaki M, Zakeri R (2014). Misdiagnosis of bland-white-garland syndrome: report of two cases with different presentations. J Cardiovasc Thorac Res.

[25] Juneja R, Shah S, Naik N, Kothari SS, Saxena A, Talwar KK (2002). Management of cardiomyopathy resulting from incessant supraventricular tachycardia in infants and children. Indian Heart J.

[26] Takemura G, Fujiwara H (2007). Doxorubicin-induced cardiomyopathy from the cardiotoxic mechanisms to management. Prog Cardiovasc Dis.

[27] Chatterjee K, Zhang J, Honbo N, Karliner JS (2010). Doxorubicin cardiomyopathy. Cardiology.

[28] Lee PC, Hwang B, Soong WJ, Meng CC (2012). The specific characteristics in children with obstructive sleep apnea and cor pulmonale. ScientificWorldJournal.

[29] Sofer S, Weinhouse E, Tal A, Wanderman KL, Margulis G, Leiberman A, Gueron M (1988). Cor pulmonale due to adenoidal or tonsillar hypertrophy or both in children. Noninvasive diagnosis and follow-up. Chest.

[30] Bannerman C, Chitsike I (1995). Cor pulmonale in children with human immunodeficiency virus infection. Ann Trop Paediatr.

[31] Verhulst SL, Van Gaal L, De Backer W, Desager K (2008). The prevalence, anatomical correlates and treatment of sleep-disordered breathing in obese children and adolescents. Sleep Med Rev.

[32] Moreira-Silva SF, Moreno LM, Dazzi M, Freire CM, Miranda AE (2012). Acute cor pulmonale due to lymphocytic interstitial pneumonia in a child with AIDS. Braz J Infect Dis.

